# Reconsidering plasmid maintenance factors for computational plasmid design

**DOI:** 10.1016/j.csbj.2018.12.001

**Published:** 2018-12-15

**Authors:** Hirokazu Yano, Masaki Shintani, Masaru Tomita, Haruo Suzuki, Taku Oshima

**Affiliations:** a Graduate School of Life Sciences, Tohoku University, 2-1-1, Katahira, Aoba-ku, Sendai 980-8577, Japan; b Department of Engineering, Graduate School of Integrated Science and Technology, Shizuoka University, 3-5-1, Hamamatsu 432-8561, Japan; c Department of Bioscience, Graduate School of Science and Technology, Shizuoka University, 3-5-1, Hamamatsu 432-8561, Japan; d Institute for Advanced Biosciences, Keio University, 14-1, Baba-cho, Tsuruoka, Yamagata 997-0035, Japan; e Faculty of Environment and Information Studies, Keio University, 5322, Endo, Fujisawa, Kanagawa 252-0882, Japan; f Department of Biotechnology, Toyama Prefectural University, 5180, Kurokawa, Imizu, Toyama 939-0398, Japan

**Keywords:** Bioinformatics, Genome design, Vector design, Synthetic biology, Plasmid persistence, Host-parasite coevolution

## Abstract

Plasmids are genetic parasites of microorganisms. The genomes of naturally occurring plasmids are expected to be polished via natural selection to achieve long-term persistence in the microbial cell population. However, plasmid genomes are extremely diverse, and the rules governing plasmid genomes are not fully understood. Therefore, computationally designing plasmid genomes optimized for model and nonmodel organisms remains challenging. Here, we summarize current knowledge of the plasmid genome organization and the factors that can affect plasmid persistence, with the aim of constructing synthetic plasmids for use in gram-negative bacteria. Then, we introduce publicly available resources, plasmid data, and bioinformatics tools that are useful for computational plasmid design.

## 1 Introduction

Plasmids are autonomously replicating DNA molecules present in microorganisms. Plasmids are also known to be mobile genetic elements that can be horizontally transferred among different organisms [1,2]. Plasmids can be considered as genetic parasites in the sense that their reproduction depends to some extent on their host and that they do not necessarily share the fate of a specific cell lineage, as they are horizontally transmissible. Plasmids have been used as primal genetic tools for exogenous DNA expression and microbial metabolic engineering. The importance of plasmid vectors has increased in recent years [3]. Currently, the plasmid genome is difficult to design computationally because the elements contained within plasmids are not conserved across plasmid groups. Additionally, a number of factors affect plasmid persistence. Understanding the key factors affecting replication and stable maintenance of plasmids in a host cell population is essential to control plasmids as synthetic vectors. Conversely, construction of synthetic vectors based on our knowledge and testing its persistence in a model host could indicate how far we have to go to understand undiscovered plasmid maintenance factors. If designed plasmids are stably maintained, it follows that the selected elements (genes, intergenic regions, etc.) play a positive role in plasmid persistence.

In this review, we summarize current knowledge of the key factors that affect plasmid persistence and then introduce publicly available resources (plasmid data and bioinformatics tools) potentially useful for designing synthetic plasmids, aiming at their use in *Escherichia coli* and other gram-negative bacteria. Reviews of the mechanisms of action of each element of a plasmid's basic function can be found elsewhere ([4,5] for partition, [6,7] for transfer, [[8], [9], [10]] for replication, and [11] for toxin-antitoxin mechanisms).

In this review, incompatibility (Inc) group classification is used to refer to plasmid groups [12,13]. Inc. groups and representative plasmid vectors relevant to gram-negative bacteria are listed in Table 1. Different plasmids belonging to the same Inc. group are incompatible and unable to be inherited in a single bacterial cell line. We note that there are also conditions, however, in which very similar or identical replicons can co-exist in the same cell [14]. Some Inc. groups defined in *Pseudomonas* are equivalent to those defined in *Escherichia coli*; for example, IncP-1, IncP-3, IncP-4, and IncP-6 are equivalent to IncP, A/C, IncQ, and IncG/U, respectively [15,16].

**Table 1:** Lists of plasmids in different incompatibility groups.

Incompatibility^a^	Representative plasmid (original host)^b^	Accession number^c^	RIP^d^	MOB^e^	MPF^e^	Host range^f^	Reference
Inc groups							
A/C_1_ (=IncA)	RA1 (*Aeromonas hydrophila*)	NC_012885	RepA	MOB_H_	MPF_F_	*Gammaproteobacteria*	[151]
A/C_2_ (=IncC)	pRMH760 (*Klebsiella pneumoniae*)	KF976462	RepA	MOB_H_	MPF_F_	*Gammaproteobacteria*	[152]
IncB/O	R3521 (*Escherichia coli*)	GU256641	RepA	MOB_P_	MPF_I_	*Escherichia*	[153]
IncD	R711b (*Providencia*)	NA	NA	NA	NA	*Escherichia, Salmonella, Proteus*	[154]
IncFI	F (*Escherichia coli*)	AP001918	RepE (for RepFIA replicon)	MOB_F_	MPF_F_	*Enterobacteriaceae, Yersiniaceae*	[155]
IncFII	R1 (*Salmonella enterica*)	KY749247	RepA	MOB_F_	MPF_F_	*Escherichia, Salmonella*	[156]
IncG/U (=IncP-6)	Rms149 (*Pseudomonas aeruginosa*)	AJ877225	RepA	MOB_P_	–	*Proteobacteria*	[157,158]
	RA3 (*Aeromonas hydrophila*)	DQ401103					
IncH	R27 (*Salmonella typhi*)	AF250878	RepHI1A, RepHI1B	MOB_H_	MPF_F_	*Enterobacteriaceae, Yersiniaceae, Erwiniaceae*	[159]
IncI	R64 (*Salmonella enterica*)	AP005147	RepZ	MOB_P_	MPF_I_	*Escherichia*, *Salmonella*, *Shigella*	[160]
IncJ	R391 (*Providencia rettgeri*)	AY090559	–	MOB_H_	MPF_F_	NA	[161]
IncK	R387 (*Shigella flexneri*)	NCTC50022		MOB_P_	MPF_I_	NA	[162]
IncL	R471 (*Serratia marcescens*)	KM406489	RepA	MOB_P_	MPF_I_	*Proteobacteria*	[163,164]
	pKOI-34 (*Klebsiella oxytoca*)	AB715422					
IncM	R69 (*Salmonella enterica*)	KM406488	RepA	MOB_P_	MPF_I_	*Proteobacteria*	[165]
	pEL60 (*Erwinia amylovora*)	NC_005246					
IncN	N3 (*Escherichia coli*)	NC_015599	RepA	MOB_F_	MPF_T_	*Escherichia*, *Klebsiella*, *Salmonella*	[166]
IncP (=IncP-1)	RK2 (*Pseudomonas aeruginosa*)	BN000925	TrfA	MOB_P_	MPF_T_	*Proteobacteria*	[167]
IncQ	RSF1010 (*Escherichia coli*)	M28829	RepABC	MOB_Q_	–	*Proteobacteria*	[168]
IncR	pKP1780 (*Klebsiella pneumoniae*)	JX424614	RepB	–	–	*Klebsiella*	[169]
IncS (=IncHI2)	R478 (*Serratia marcescens*)	BX664015	RepHIA	MOB_H_	MPF_F_	*Serratia*	[170]
IncT	Rts1 (*Proteus vulgaris*)	AP004237		MOB_H_	MPF_F_	*Proteus*, *Citrobacter*	[171]
IncV	R753 (*Escherichia coli*)	NCTC50521 (planned)	NA	NA	NA	*Proteus*	[172]
IncW	R388 (*Escherichia coli*)	NC_028464	RepA	MOB_F_	MPF_T_	*Salmonella*, *Escherichia*, *Providencia*	[173]
IncX	R6K (*Escherichia coli*)	NCTC50005	π	MOB_P_	MPF_T_	*Enterobacteriaceae*	[174]
IncY	P1 (*Escherichia* virus)	AF234172 (phage P1 *mod749*::IS*5 c*1–100)	RepA	–	–	*Enterobacteriaceae*	[175,176]
	pMCR-1-P3 (*Escherichia coli*)	KX880944					
IncZ	pEI545 (*Klebsiella pneumoniae*)	M93064 (partial)	RepA	NA	NA	*Klabsiella*	[162]
PromA	pMRAD02 (*Methylobacterium radiotoleran*s)	NC_010509	RepA	MOB_P_	MPF_T_	*Proteobacteria*	[[177], [178], [179]]
	pIPO2 (gunknown)	AJ297913					
	pSN0729-62 (gunknown)	AP018705					
IncP-9	pM3 (*Pseudomonas putida*)	AF078924 (partial)	Rep	MOB_F_	MPF_T_	*Pseudomonas, Escherichia*	[180,181]
	NAH7 (*Pseudomonas putida*)	NC_007926					

Representative cloning vectors							
Not assigned	pUC18/19, pET, pBluescript	L09136	pMB1 copy-up type, pMB1 type, pMB1 copy-up type	–	–	*Escherichia*	[175] Merck Millipore[182]
Not assigned	pACYC148	X06403	p15A type	–	–	*Escherichia*	[183]
Not assigned	pBBR1MCS (*Bordetella bronchiseptica*)	NC_025015	pBBR1 Rep	MOB_V_	–	*Proteobacteria*	[184]
Not assigned	pSC101 (*Salmonella enterica*)	NC_002056	RepA	MOB_Q_	–	*Salmonella, Escherichia*	[185]
Not assigned	pABC1 (*Rhizobium etli*)	KY031728	p42b RepC	–	–	*Rhizobiales*	[44]
Not assigned	pME6041 (*Pseudomonas* sp.)	AF118812	pVS1 RepA	–	–	*Proteobacateria*	[65]

a Several Inc. groups are identical; e.g. IncG = IncU.

b Representative plasmids are listed based on Lawley et al. [186].

c Accession numbers in National Center for Biotechnology Information (NCBI) GenBank and RefSeq (https://www.ncbi.nlm.nih.gov/Sequin/acc.html) and Wellcome Sanger Institute (prefix “NCTC” https://www.sanger.ac.uk/resources/downloads/plasmids).

d Names of replication initiation protein (RIP). “NA” indicates that the nucleotide sequences of the plasmid are not available.

e Classification of MOB classes and MPF types is based on Smillie et al. [132] and Guglielmini et al. [187]. “-” indicates that the genes involved in conjugation have not been detected, whereas “NA” indicates that the nucleotide sequences of the plasmid are not available.

f Plasmid host range determined based on genome sequencing projects (hosts in which a plasmid has been found) and/or filter mating assays.

g Original hosts are unknown because exogenous plasmid capturing was used.

## 2 Key Factors in Plasmid Design

Based on recent progress in plasmid biology and bioinformatics, we consider three factors that should be taken into account to design a synthetic plasmid (Table 2): 1) plasmid gene content; 2) interaction with host (host factors and fitness cost imposed by plasmids); and 3) constraints in genome (size, sequence composition [e.g., G + C content, oligonucleotide composition, and codon usage], and gene direction). These factors are described in detail in the subsequent sections.

**Table 2:** Key factors in the construction of a plasmid vector.

Factors	Notes (what should be considered)
1. Plasmid gene content	Include a set of plasmid core genes.
	Include selection marker or a toxin-antitoxin system to prevent generation of plasmid-free cells.
	Include *cis*-elements, such as centromere-like site and resolution site.
2. Interaction with host	Select a basic replicon that has evolved in species closely related to a model host.
	Transcriptional regulator or NAPs (H-NS homologs) for plasmid genes could reduce the fitness cost imposed by the plasmid.
3. Constraints in genome	The G + C content of the plasmid should match that of the host.
	Highly expressed essential genes should be on leading strands.

### 2.1 Plasmid Gene Content

#### 2.1.1 Defining the Plasmid Core

Plasmids show gene content variations, even within the same Inc. group [17]. Thus, plasmids are likely to experience gene gain and loss over evolutionary time [18,19]. A comparative analysis of closely related taxa can categorize a genome into two parts: (i) “core” genes conserved in all members within a defined group (e.g., bacterial species, Inc. group, etc.), and (ii) “noncore” genes absent in some members within the group. Being a core gene does not necessarily mean that the gene positively contributes to plasmid maintenance in particular hosts, but suggests that the gene sets have co-evolved together since the divergence from the most recent common ancestor. The long-term co-evolution of core genes can result in the formation of an operon with a coordinated regulatory system that balances the efficiency of horizontal and vertical transmissions [[20], [21], [22]]. These core genes may be linked together upon construction of a vector. A recent analysis of recombination tracts in the plasmid core genome highlighted a block of evolutionarily linked genes [23]. These findings also suggest that the plasmid core undergoes recombinational allelic exchange within the group at an evolutionary time scale.

Core and noncore genes can be identified by homologous gene clustering for a defined plasmid group, e.g., using all-against-all protein sequence comparisons with BLASTP [24]. We previously found that homolog clusters specific to each of the six Inc. groups (F, H, I, N, P-1, and W) (Table 1) were involved in plasmid replication, partition, and transfer [17]. Based on the BLASTP (*E*-value <1e−5) comparison, replication initiation (Rep) proteins for the six Inc. groups (RepB and RepE for IncFI, RepAfor IncFII, RepHIA for IncH, RepZ for IncI, RepA for IncN, TrfA for IncP-1, and RepA for IncW) formed distinct homolog clusters (exceptions were RepB and RepHIA, which formed a single homolog cluster) and were conserved in all members within each of the Inc. groups.

In the IncP-1 group, TrfA (replication initiation protein), KlcA (antirestriction protein), KlcC (KorC transcriptional repressor), KleE (stable inheritance protein), KfrA, IncC (ParA homolog), and KorB (ParB homolog) were conserved in all 22 plasmids analyzed [17] and thus deemed as the plasmid core. Surprisingly, a homolog cluster for DNA transfer (*tra*, *trb*), postsegregational cell killing (*parDE*, *relBE*), multimer resolution (*parA*), and regulatory protein (*korA*), which was shown to support plasmid maintenance ([25]), was not identified as the plasmid core. The absence of genes suggests lineage-specific gene loss or nonorthologous gene displacement during plasmid evolution in nature [18,19].

#### 2.1.2 Functional Modules Comprising a Plasmid

**Fig. 1:**
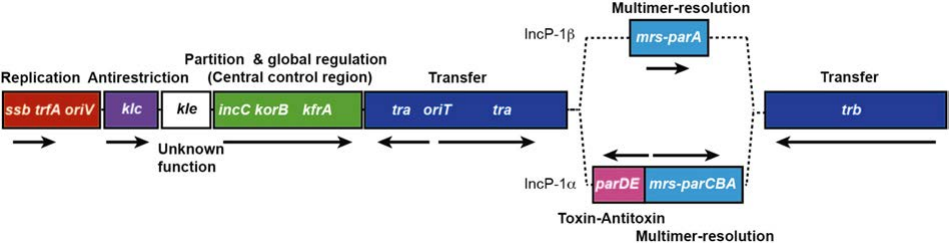
Modular structure of the plasmid genome. An example of the IncP-1 plasmid genome is shown. The replication module is the only element essential for plasmid maintenance. The other modules assist in plasmid maintenance. IncP-1β and IncP-1α are lineages of IncP-1 plasmids. Horizontal arrows indicate the direction of transcription. Each functional module is regulated by a transcriptional regulator encoded in the module block or global regulators encoded in the central control region [188,189].

Gene products, which contribute to plasmid maintenance in bacterial hosts, require *cis*-acting sites to elicit their functions. In this review, a functional module is defined as a pair of gene products and its acting site on a plasmid. Each functional module often contains its own regulatory function. In such cases, the elements of each functional module should not be separated upon construction of a synthetic plasmid. Below, we briefly describe the features of representative functional modules comprising a plasmid, i.e., replication module, partition module, toxin-antitoxin module, multimer resolution module, DNA transfer module, and antirestriction module. Plasmid genomes are often considered an assembly of these functional modules (Fig. 1). Plasmid functional modules are potential sources for biological parts for synthetic biology projects, such as BioBrick [26] and SEVA [27].

##### 2.1.2.1 Replication Module

**Fig. 2:**
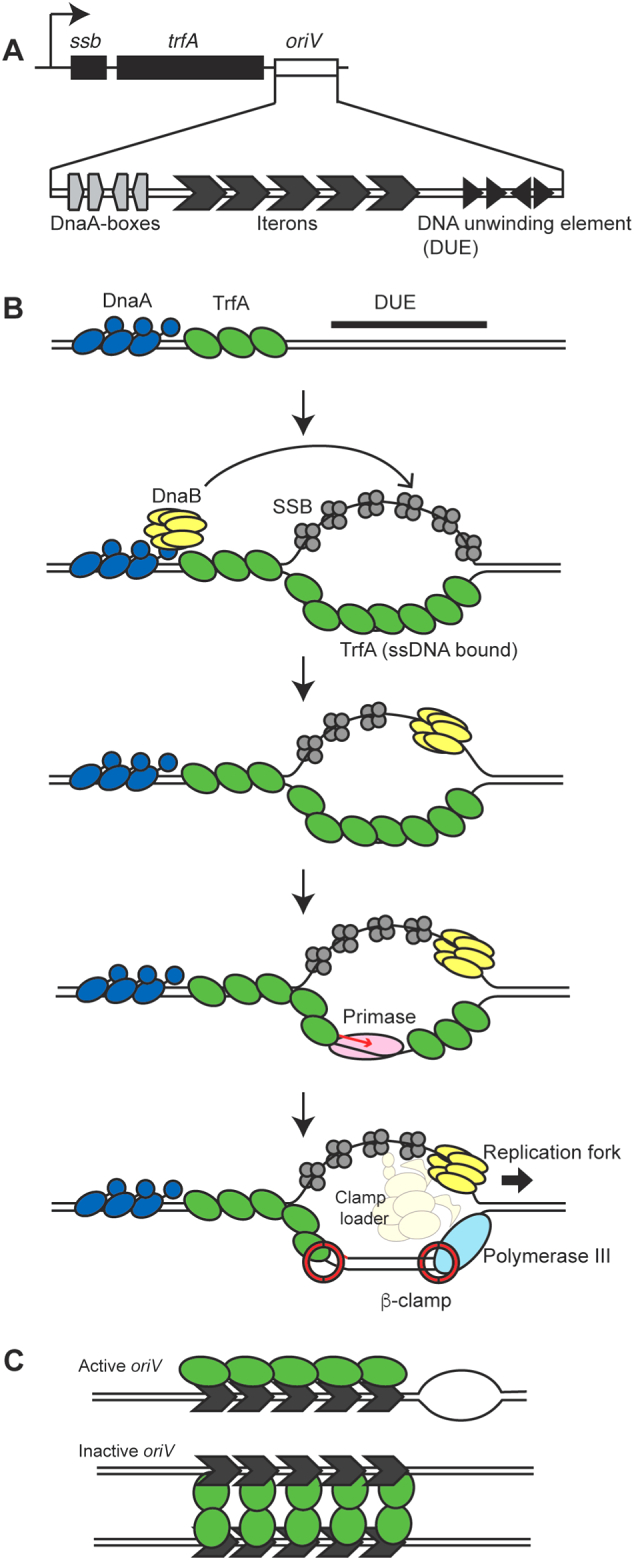
Replication module. (A) Replication module of IncP-1 plasmids. *Ssb* encodes a single-strand DNA binding protein. *trfA* encodes the Rep protein. (B) Replication initiation of IncP-1 plasmid. Monomeric TrfA bound to iterons opens base pairs in the DUE, and then host DnaA or TrfA itself recruits DnaB onto the unwound DUE. TrfA molecules bind to single-stranded DNA to assist replisome assembly on one strand via direct interaction with β-clamp, starting unidirectional replication. Lagging strand synthesis is not shown for simplicity. Illustration follows [8], with minor modifications. (C) Replication inhibition by Rep protein dimer. Paired *oriV* can dissociate via proteolysis or an increased Rep protein monomer to dimer ratio [32].

Plasmids can carry two types of replication origins; one is a vegetative origin (*oriV*), and the other is a transfer origin (*oriT*). In this section, we describe the replication module that uses *oriV.* In ColE1-type plasmids , the replication module consists of *oriV* and genes for two noncoding RNAs (RNA I and RNA II) and Rop protein, which are produced from near the *oriV* [28]. RNA II is converted to primer RNA (thus acts as an initiator of replication), whereas RNA I and Rop protein cooperatively inhibit RNA II maturation (thus act as inhibitors of replication). The copy number of ColE1-type plasmids is maintained at around 10–15 copies/cell [9]. This type of replicon has been used as a cloning vector, including pUC and pET vectors (Table 1). For pUC vectors, deletion of the Rop protein gene and a point mutation in RNA II result in a dramatic increase in copy numbers (500–700 copies/cell) [29]. The replication modules of so-called iteron-containing plasmids, e.g., RK2 and R6K (Table 1), consist of a replication initiation protein (Rep protein) gene and *oriV*, which are in general located next to each other on the plasmid (Fig. 2A). *oriV* contains a Rep protein-binding region (iterons), host DnaA-binding region (DnaA-boxes), and DNA unwinding elements (DUE), which are motifs in an A + T-rich region within *oriV* [8]. Rep proteins act as both initiators and inhibitors of replication [8,28,30]. Purified Rep proteins are mostly dimeric, whereas only monomeric Rep protein is active in unwinding DUE (Fig. 2B). DnaB helicase is loaded onto unwound DUE via either a host DnaA-dependent or -independent manner. Rep protein can also bind a specific strand of unwound DUE and assists replisome assembly on one strand via direct interaction with β-clamp, leading to unidirectional replication [31]. Dimeric Rep proteins prevent *oriV* melting by pairing iterons in a phenomenon called handcuffing (Fig. 2C) [[32], [33], [34]] An increased monomer to dimer ratio dissociates the paired iterons [32,33].

A Rep protein mutant of R6K π protein (pir-116 allele) [35] lacks replication inhibition activity (unable to form dimer) and has been used to increase the vector copy number only in specific *Escherichia coli* cloning hosts [36]. The copy number of iteron-containing plasmids is normally 1–8 copies/chromosome [37]. Replication initiation from *oriV* usually requires host DnaA.

Theta-type replication can be either uni- or bidirectional, whereas rolling circle replication is unidirectional [9]. Strand-displacement replication of IncQ plasmids is bidirectional [10]. In most plasmids, theta-type replication is unidirectional (exceptions include the linear *Streptomyces* plasmid [38]), and there is no replication termination site (exceptions include the plasmid R6K [9,39]).

##### 2.1.2.2 Partition Module

**Fig. 3:**
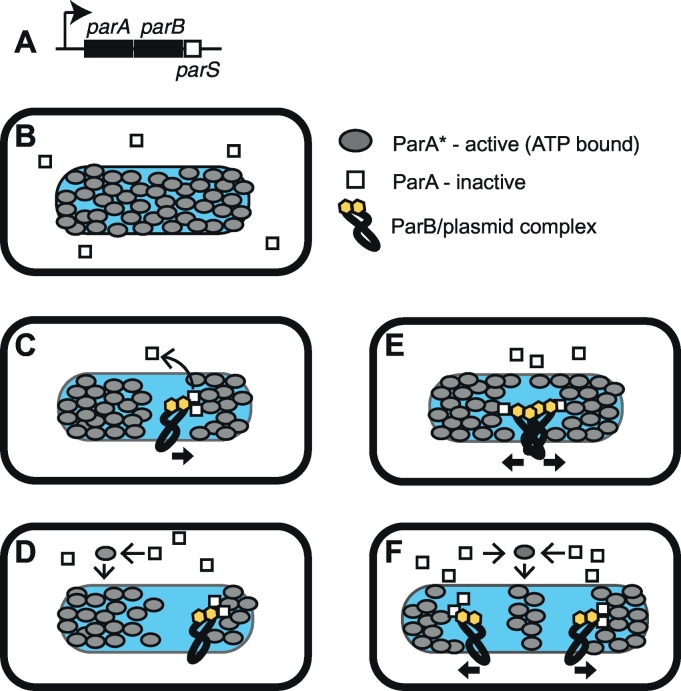
Partition module. (A) *par* locus of P1 prophage (type I partition system). *parA* encodes Walker-type ATPase. *parB* encodes centromere-binding protein. *parS* is centromere. (B-F) Diffusion-ratchet model of plasmid motion. (B) ParA has two states: ParA*, the ATP-bound form, active in binding DNA; ParA, other forms inactive in binding DNA. ParB binds to *parS*. For simplicity, only two ParB molecules are shown. (C) ParB binding to ParA* activates ATP hydrolysis by ParA. ParA is released from the nucleoid. (D) ParA slowly exchanges ADP with ATP, then returns to the nucleoid surface. (E) When replicated plasmid copies are present in close proximity, a ParA*-free area is generated between them. Each ParB/plasmid complex diffuses until finding its closest ParA*; thus, their interactions are repulsive. (F) ParB/plasmid complexes are pulled to ParA*-dense areas at opposite ends, following the gradient of ParA*. Illustration follows [5], with modifications.

Naturally occurring low copy number plasmids have active segregation mechanisms to avoid plasmid loss upon cell division. These mechanisms are equivalent to the function of the spindle apparatus in a eukaryotic cell [40]. Currently, three types of segregation mechanisms have been proposed [4,5]. Each system consists of a centromere site (often referred to as *parS*), centromere-binding protein (ParB), and motor protein (ParA). Here, we call a set of the genes and sites for those elements a partition module. A centromere site is generally located directly upstream or downstream of *par* genes [41,42]. The segregation mechanism employed by the type I partition system is shown in Fig. 3. In P1 prophage (Table 1), the partition module consists of the *parA-parB* operon and its downstream *parS* region, which contains multiple ParB binding sites and a host IHF binding site [41]. ParA molecules bound to ATP (ParA*) can bind to DNA non-specifically and thus localize to the nucleoid. The binding of ParB to ParA* activates ATP hydrolysis by ParA, disrupting the ability of ParA to bind to DNA and releasing it from the nucleoid. Once ParA* is cleared, the ParB/plasmid complex diffuses through the nucleoid until it makes contact with ParA*. ParB/plasmid complexes in close proximity generate repulsive forces as they clear ParA* between them. Therefore, replicated plasmid copies are respectively pulled to the opposite ParA*-dense area following the gradient of ParA*(Fig. 3) [5].

*repABC* family plasmids from *Alphaproteobacteria* [43] carry a replication module and partition module in the same locus (*repABC*), and the *repABC* locus has been used as a vector core for certain types of vectors [44].

##### 2.1.2.3 Toxin-antitoxin (TA) Module

Because plasmids are not tightly connected to the chromosome that carries genes essential for bacterial hosts, cell division can generate plasmid-free cells. If the plasmid is lost upon cell division, the plasmid-free cells, which grow faster than plasmid-containing cells can show an increase in relative population size. This phenomenon can be suppressed by a mechanism called postsegregational cell killing, wherein plasmids produce both stable toxin and unstable antitoxin that counteracts the toxin; plasmid loss results in increased toxin levels in the cells, leading to growth inhibition or cell death of plasmid-free cells [45]. The genetic module responsible for this phenomenon is called the TA module. TA modules can be categorized into six groups according to the mechanism of action [11,46,47]. The first TA system discovered is the *hok/sok* system of plasmid R1 (Table 1) [45], currently classified as a type I TA module, in which *sok* encodes an antisense RNA that inhibits the translation of the Mok protein, a regulator for Hok toxin, which generates pores in the cell wall. The *hok/sok* module of plasmid R1 was applied to improve vector maintenance in the chemostat [48]. The *ccdA/ccdB* module discovered in plasmid F (Table 1) [49] has also been used in biotechnology. The CcdB toxin inhibits the function of the host DNA gyrase. The *ccdB* gene has been used as a counter selection marker [50], e.g., in Gateway cloning technology and allele replacements in the chromosome [51,52]. By separating the toxin element and the antitoxin element of a TA module into the chromosome and vector, respectively, StabyCloning technology (Delphi Genetics) enables stable maintenance of a protein-expression vector in the *Escherichia coli* cell population.

##### 2.1.2.4 Multimer Resolution Module

Replicated plasmid copies can recombine into a dimer to multimer via homologous recombination; this negatively affects plasmid partition. Naturally occurring plasmids encode a genetic module to resolve this problem. Small mobilizable plasmids use host-encoded proteins (site-specific recombinases XerC and XerD and accessory proteins PepA and ArgR [53]) for their dimer resolution, and the plasmids carry only a *cis*-acting resolution site (e.g., *cer* for ColE1 and its related plasmids, *psi* for pSC101 [[54], [55], [56]]). Larger self-transmissible plasmids, e.g., IncP-1 plasmids, carry a host-independent multimer resolution module consisting of a site-specific recombinase (resolvase) gene and a resolution site that also functions as a regulatory region for the resolvase gene [57]. Lack of a resolution module on the plasmid appears to be eventually compensated for by the acquisition of a functionally equivalent cointegrate-resolution system of a Tn*3* family transposon, according to observations in experimental evolution [58].

##### 2.1.2.5 DNA Transfer Module and Antirestriction

Conjugative transfer is an important feature of plasmids that enables them to spread genetic information among bacteria (current paradigms for conjugation are summarized in [7]). There are self-transmissible plasmids, mobilizable plasmids, and nonmobilizable or nontransferrable plasmids [59]. The self-transmissible plasmids carry all the gene sets and a *cis*-acting site (*oriT*) required for mating pair formation and DNA processing, whereas mobilizable plasmids carry the genes and site only for DNA processing.

The Ti plasmid of genus *Agrobacterium* carries two types of DNA transfer modules: (i) *tra/trb* operons for DNA transfer between bacteria and (ii) a *vir* operon for DNA transfer between bacteria and plants [60]. Plasmids from gram-negative bacteria generally use a type IV secretion system for DNA transport, whereas some plasmids from gram-positive bacteria use different DNA transport mechanisms [7,61,62].

Non-self-transmissible plasmids, including IncQ plasmids represented by RSF1010 (Table 1), can be mobilized by self-transmissible plasmids, e.g., by the IncP-1 plasmid RK2 [63]. *oriT* has been embedded in some cloning vectors to mobilize the vectors into various hosts for which transformation methods have not been established or are inefficient [[64], [65], [66]].

Plasmid gene content analysis revealed that the complete gene set responsible for self-transmissibility is not necessarily conserved across members of each self-transmissible plasmid group, e.g., IncW and IncP-1 [17]. Interestingly, a gene encoding an antirestriction protein, which blocks the host's restriction system upon plasmid entry into new hosts, was found to be an element of the plasmid core in IncP-1 and IncW [17]. ArdB, KlcA_,_ ArdA, and ArdC homologs can confer antirestriction against the host's type I restriction-modification system [[67], [68], [69]]. These antirestriction genes may be important for transfer of synthetic plasmids between different bacterial lineages.

#### 2.1.3 Testing the Functionality of Functional Modules

To evaluate the contribution of each functional module to plasmid maintenance, a set of highly unstable broad-host-range plasmid vectors based on the RK2 replicon of the IncP-1 group has been constructed [70]. For example, the functionality of the partition module of a IncU plasmid (Table 1), the chromosome partitioning system of *Pseudomonas aeruginosa*, and the *hipAB* TA system of the *Paracoccus kondratievae* plasmid have been confirmed using these vectors [70].

#### 2.1.4 Selection Markers

Antibiotics have traditionally been used to select plasmid-containing cells in culture in the laboratory. Mainly for biosafety reasons, various antibiotic marker-free selection approaches have been developed [3,71,72]. Some of the tricks used in such approaches are based on plasmid-derived elements: for example, the RNA I and II of plasmid ColE1 have been used in an antibiotic-free host-vector system [73].

### 2.2 Interactions with the Host

Early biochemical studies and recent experimental evolution studies have suggested the importance of host factors and fitness cost for plasmid carriage. These factors are discussed below.

#### 2.2.1 Host Factors

Most plasmids require the host's replication initiator DnaA and DNA helicase encoded by the host chromosome or plasmid itself upon replication initiation from *oriV* [9]. Whether plasmids can load DNA helicase at the *oriV* using DnaA or plasmid's Rep protein determines the capability of plasmid replication in the host cells and their replication host range [74,75]. Nucleoid-associated proteins (NAPs), such as histone-like nucleoid-structuring protein (H-NS) are known to make the DNA structure more compact [76]. Moreover, chromosomally encoded NAPs have been shown to affect gene expression from the IncP-7 plasmid pCAR1 [77,78].

#### 2.2.2 Fitness Cost Imposed by Plasmids

When plasmids are introduced to novel hosts, plasmids initially impose a fitness cost on the hosts and are thus not necessarily stably maintained, particularly in laboratory systems [72,79,80]. It should be noted that in nature, plasmids can persist without positive selection, despite their detectable costs in laboratory systems [72]. Resequencing of experimentally evolved plasmid-host pairs in several independent studies suggests that initial interactions between the host gene and plasmid gene are unfavorable for the host's growth. Although the cause of the cost can be different among plasmid-host pairs, reduced interaction appears to improve host growth and plasmid maintenance [[81], [82], [83]]. These observations are consistent with the complexity hypothesis, which states that the number of interaction partners predicts the horizontal transfer ability of a gene [84,85]. Using a series of antibiotic resistance genes as a model of horizontally acquired genes, Porse et al. [86] demonstrated that physiological interaction of the gene products with hosts imposes a greater cost than nucleotide signals, e.g., G + C content and codon usage. The cause of costs may be relevant to the interactions summarized elsewhere [87] (e.g., disruptive interactions with cellular networks). Currently, it is difficult to predict which interactions negatively affect host fitness and plasmid persistence for an arbitrarily chosen host-plasmid pair. Experimental evolution may help reduce the fitness cost imposed by a synthetic plasmid.

Transcriptome disturbance by a plasmid in a new host is initially high, but will be reduced during fitness cost amelioration [81,82]. Moreover, plasmids encoding H-NS-like stealth protein reduce their fitness cost probably by silencing transcriptional activities of genes in the A + T rich region through the binding of H-NS-like proteins [78,88]. In contrast to smaller or nontransmissible plasmids, larger and transferable plasmids carried multiple NAP genes [89,90]. Three different NAPs encoded on plasmid pCAR1 are involved in plasmid stability and its conjugation in the host cells [91]. Therefore, minimizing unnecessary transcription may be important for minimizing the cost imposed by plasmids.

### 2.3 Constraints in the Genome

Bioinformatics analysis revealed constraints in plasmids with respect to size, sequence composition (G + C content, oligonucleotide composition, and codon usage), and gene direction. These features may be a result of plasmid-host co-evolution, which can stabilize plasmids in host cell populations. It is important to note that the sequence composition can vary among genes/segments within a plasmid/genome [23,92,93].

#### 2.3.1 Size Constraint

**Fig. 4:**
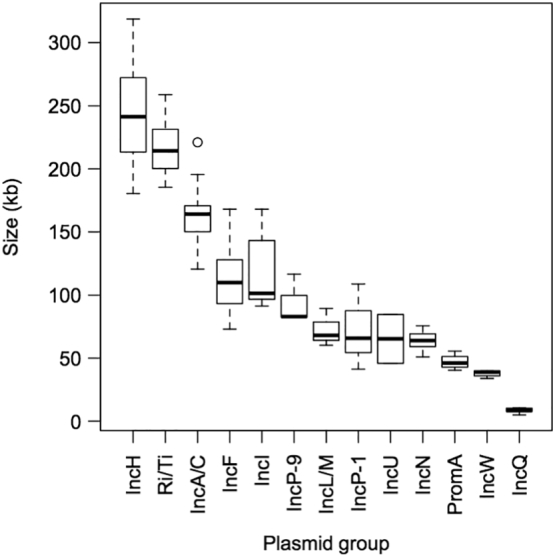
Box-and-whisker plots displaying the distributions of plasmid sizes (kb) for various plasmid groups based on a five-number summary (minimum, 25th percentile, median, 75th percentile, and maximum). Outliers are plotted as open circles. Data are from Suzuki et al. [17].

The size distribution of sequenced plasmids available in public databases has been studied. For example, sizes for the 4602 completely sequenced plasmids ranged from 744 bp to 2.58 Mb with a mean value of 80 kb, and the mean value of sizes for mobilizable plasmids was smaller than that for transmissible plasmids [59]. Among the 92 plasmids from the IncF, IncH, IncI, IncN, IncP-1, IncW, A/C, IncL/M, IncP-9, IncQ, IncU, PromA, and Ri/Ti groups used in Suzuki et al. [17], sizes for the non-self-transmissible IncQ plasmids (median size of 8.7 kb) were smallest. Among the self-transmissible plasmids belonging to the six Inc. groups F, H, I, N, P-1, and W, the median value of sizes (kb) was highest for the IncH group (241 kb), followed by those of the IncF (110 kb), IncI (101 kb), IncP-1 (66 kb), IncN (64 kb), and IncW (39 kb) groups (Fig. 4). Because each plasmid group has specific range of genome sizes, it may be important to keep plasmid size in the appropriate range considering the replicon type used in the vector.

Plasmid size may be associated with copy number. For 11 plasmids found in *Bacillus thuringiensis* strain YBT-1520, the plasmid sizes (ranging from 2 to 416 kb) and the copy numbers determined by quantitative polymerase chain reaction (ranging from 1.38 to 172) were negatively correlated [94]. Plasmid F, a member of IncF (median size: 1110 kb), is present at 1 or 2 copies per chromosome, whereas the copy number of RK2, a member of the IncP-1 group (median size: 66 kb), is 3–5 copies/chromosome (in the presence of large replication protein TrfA1), or 1–2 copies/chromosome (without TrfA1) [95]. Plasmid pR28, a member of the IncP-9 group (median size: 83 kb) has a copy number of 1.6–3.7/chromosome [58]. Copy numbers of the IncQ mobilizable plasmids (median size: 8.7 kb) are 10–16/chromosome [96]. Copy numbers of ColE1-related plasmids are 20–44/chromosome [87,97]. Conlan et al. (2014) determined the copy numbers of plasmids in *Enterobacteriaceae* (3 A/C, 6 IncF, 1 IncHI2, 8 IncN, and other plasmids) from the average sequence coverage (depths of PacBio and MiSeq reads) of each plasmid relative to that of the chromosome and showed that copy numbers were 1–3/chromosome [98]. Plasmid copy number estimates can vary, depending on bacterial growth conditions and DNA extraction methods used [97,99]. Therefore, copy number data should be interpreted carefully. To the best of our knowledge, there is no database that catalogs the plasmid copy numbers in various hosts under the same experimental conditions. The elucidation of clear features of plasmid maintenance functions associated with copy number or replicon type requires further investigation.

#### 2.3.2 G + C Content

**Fig. 5:**
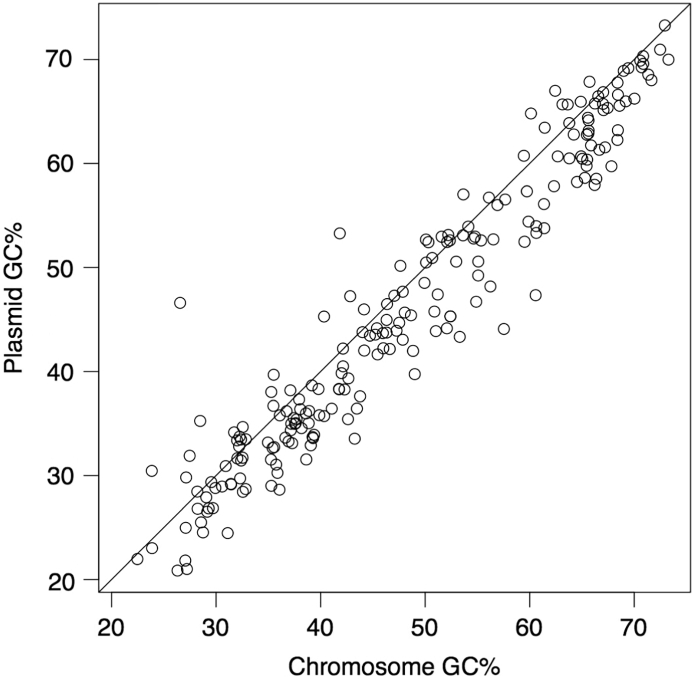
Plot of G + C contents of 209 plasmids and their host chromosomes. Each point represents a plasmid-chromosome pair from 209 prokaryotes. To minimize the bias in the numbers of sequenced organisms and replicons available in public databases (e.g., thousands of genome projects for *Escherichia coli*, and multireplicons for *Borrelia* species), RefSeq data for completely sequenced prokaryotes that consist of one chromosome and plasmid were retrieved on April 17, 2017 from a list of all selected representative prokaryotic genomes (ftp://ftp.ncbi.nlm.nih.gov/genomes/GENOME_REPORTS/prok_representative_genomes.txt). The G + C contents of plasmids tend to be lower than (and are correlated with) those of the host chromosomes.

G + C contents vary widely among bacterial genomes, putatively reflecting a balance among biases generated by mutation and selection [100]. Because bacterial genomes have small regions of noncoding DNA and more protein-coding constraints on first- and second-codon positions than on third-codon positions, most of the variations are due to synonymously variable third-codon positions [101,102]. Growth rate experiments in *Escherichia coli* and *Caulobacter crescentus* showed that decreased genic G + C contents at synonymous sites have negative effects on bacterial fitness when gene expression levels are induced [100,103]. Previous studies have reported that small bacterial genomes tend to exhibit low G + C contents, with some exceptions [104], and that intracellular symbionts, such as plasmids and phages, tend to have lower G + C contents than their hosts [92,105,106]. For the 209 plasmids and their host chromosomes, the G + C contents are highly correlated, and in 164 (78.5%) of cases, plasmids had lower G + C contents than their hosts (Fig. 5). Possible explanations for the lower G + C contents of plasmids than those of hosts include the selection of plasmids that tolerated gene silencing by host H-NS [88,107] and reduced nucleotide synthesis costs [105]. Thus, it may be important that the G + C contents of synthetic plasmids match those of the host chromosomes.

#### 2.3.3 Oligonucleotide Composition

The composition of oligonucleotides, such as di-, tri-, and tetra-nucleotides (also known as *k*-mers, such as 2-, 3-, and 4-mers), has been studied for the characterization and classification of various organismal genomes [108,109]. Plasmids have oligonucleotide compositions similar to those of their host chromosomes [93,109]. The compositional similarities of plasmids and their hosts suggests that plasmids have acquired hosts' nucleotide compositions due to amelioration by host-specific mutational biases [110]. Thus, possible plasmid-host pairs are predictable based on the similarity of their oligonucleotide compositions [17]. Earlier studies investigated sequence motifs in the IncP-1 plasmids RK2 [111] and R751 [112] and suggested that some sequence motifs (e.g., tetranucleotide and hexanucleotide palindromic sequences acting as restriction-modification sites) may have been eliminated from plasmids through natural selection. Computational analysis of oligonucleotide compositions has been used to identify novel regulatory DNA sequence motifs [113], some of which may be important for stable plasmid maintenance.

#### 2.3.4 Codon Usage

Synonymous codon usage, tRNA abundance, and ribosome density are critical factors for protein synthesis [[114], [115], [116]]. Codons can affect translation efficiency, protein folding, and mRNA stability [117].

In bacteria such as *Escherichia coli* and *Bacillus subtilis*, highly expressed genes (e.g., those encoding translation elongation factors and ribosomal proteins) tend to preferentially use a subset of synonymous codons that are best recognized by the most abundant tRNA species [118,119]. This is considered evidence of natural selection on synonymous codon usage for translational efficiency and/or accuracy (also called translational selection) [114,120]. Previous studies have indicated that the strength of translational selection on chromosomes varies among bacteria and that fast-growing bacteria with more rRNA and tRNA genes are subjected to strong selection pressure [102]. The strength of translational selection also varies among replicons within the same organism; for example, in *Sinorhizobium meliloti*, codon usage of the chromosome and plasmids pSymB and pSymA reflects their importance for competitive cell growth and expression during the free-living stage of the organism [121].

The codon usage of plasmids is not always similar to that of the host chromosome. Measuring the distance between the codon usages of pairs of *Agrobacterium tumefaciens* replicons (circular and linear chromosomes and plasmids pAt and pTi) revealed that the distances between chromosomes and plasmids are larger than the distances between the two chromosomes (circular and linear) or the two plasmids (pAt and pTi) [122]. For each pair of three *Agrobacterium* species (*Agrobacterium tumefaciens* C58, *Agrobacterium vitis* S4, and *Agrobacterium radiobacter* K84), codon usages of their plasmids, with varying gene contents, are more similar than codon usages of their chromosomes [123]. It remains unclear whether codon usage influences stable plasmid maintenance in hosts, and the fitness cost imposed by plasmids is still unknown.

#### 2.3.5 Gene Direction

Bioinformatics algorithms based on replication strand biases, such as GC skew, defined as (C - G)/(C + G), have been used to predict replication origin and terminus in bacterial chromosomes and plasmids [[124], [125], [126]]. The degree of GC skew is different between plasmids with and without rolling circle replication and is correlated between plasmids and chromosomes of bacteria, suggesting that replication-related mutation and selection determine the strength of GC skew for replicons within the same host [127]. Previous studies reported that coding sequences (5′ to 3′ orientation) in the bacterial chromosome are preferentially located on the template strands for lagging-strand synthesis (also simply referred to as leading strands [128]), and this codirectional bias of replication and transcription is further enriched in essential and/or highly expressed genes [[128], [129], [130], [131]]. It remains unclear whether gene expressivity and essentiality influence the orientation bias of plasmid genes; however, it may be better to carry important genes on the leading strand of the synthetic plasmids, following the trend in the chromosome.

## 3 Publicly Available Resources

Comparative sequence analyses of closely related plasmids with different features, such as replication, maintenance, transfer, and host range, can provide hypotheses regarding genetic determinants of these plasmid features. Over the past 10 years, plasmid sequence data have been dramatically increased, and convenient bioinformatics tools have been developed to manage and analyze the data. These resources are briefly described in this section.

### 3.1 Plasmid Sequence Data

High-throughputDNA sequencing has generated a large amount of plasmid sequences, which can be retrieved from the International Nucleotide Sequence Database Collaboration or INSDC: DDBJ, EMBL-EBI, and NCBI (http://www.insdc.org). As of 2010, the 1,730 complete plasmid sequences in GenBank were obtained from plasmid-sequencing projects (62%) and microbial genome projects (38%) [132]. In 2015, Shintani et al. [59] used the NCBI database to review the classification of completely sequenced plasmids based on their host taxonomy and features of replication and transfer. Based on the NCBI list of plasmid sequences, downloaded on November 22, 2018 from ftp://ftp.ncbi.nlm.nih.gov/genomes/GENOME_REPORTS/plasmids.txt, there are currently 14,309 complete sequences of plasmids, of which 115 are from Eukaryota, 196 from Archaea, and 13,998 from Bacteria (8,538 from Proteobacteria, 3,206 from Firmicutes, 747 from Spirochaetes, and 606 from Actinobacteria).

Because INSDC databases covering all available nucleotide data are not always well curated and structured, secondary databases have been developed. For example, the ACLAME database (http://aclame.ulb.ac.be) [133] has been developed and used to investigate the general features of sequenced plasmids, such as their distribution per host species [134]. Orlek et al. [135] presented a curated dataset of complete Enterobacteriaceae plasmids compiled from the NCBI database (https://figshare.com/s/18de8bdcbba47dbaba41). The web servers PLSDB (https://ccb-microbe.cs.uni-saarland.de/plsdb/) [136] and pATLAS (http://www.patlas.site) [137] provide a more comprehensive collection of bacterial plasmids retrieved from the NCBI nucleotide database.

### 3.2 Bioinformatics Tools

Bioinformatics tools can be used to design synthetic plasmids by searching, assembling, and adjusting key factors, including functional module (genes and *cis*-element) and genome constraints. Table 3 lists bioinformatics tools for plasmids with their URLs.

**Table 3:** List of bioinformatics tools for plasmid sequence analysis and vector design.

Usage	Name	URL
*Viewing/editing plasmid sequences*		
	ApE (A plasmid Editor)	http://biologylabs.utah.edu/jorgensen/wayned/ape/
	SnapGene	http://www.snapgene.com
	Benchling	https://benchling.com/molecular-biology

*Designing vectors by assembling modules*		
	SEVA-DB	http://seva.cnb.csic.es

*Reconstructing plasmids from sequencing reads or assembly graphs*		
	PLACNET	https://sourceforge.net/projects/placnet/
	PLACNETw	https://castillo.dicom.unican.es/upload/
	plasmidSPAdes	http://cab.spbu.ru/software/spades/
	Recycler	https://github.com/Shamir-Lab/Recycler
	PlasmidTron	https://github.com/sanger-pathogens/plasmidtron

*Detecting plasmids in assembled contigs*		
	PlasmidFinder	https://cge.cbs.dtu.dk/services/PlasmidFinder/
	cBar	http://csbl.bmb.uga.edu/~ffzhou/cBar
	PlasFlow	https://github.com/smaegol/PlasFlow
	MOB-suite	https://github.com/phac-nml/mob-suite

*Detecting plasmids in unassembled reads*		
	PlasmidSeeker	https://github.com/bioinfo-ut/PlasmidSeeker

Recent studies have developed bioinformatics tools for detecting plasmids from whole genome sequencing data, including cBar [138], PlasmidFinder [139], PLACNET [140], plasmidSPAdes [141], Recycler [142], PlasFlow [143], PlasmidTron [144], PlasmidSeeker [145], and MOB-suite [146]. PLACNETw is a web tool based on PLACNET [147]. These tools can be used for plasmid reconstruction by assembling sequencing reads or from de novo assembly graphs (PLACNET/PLACNETw, plasmidSPAdes, Recycler, and PlasmidTron) or for plasmid detection in assembled contigs (PlasmidFinder, cBar, PlasFlow, and MOB-suite) or in unassembled reads (PlasmidSeeker). cBar, PlasFlow, PlasmidTron, and PlasmidSeeker perform *k*-mer-based analyses to detect plasmids. plasmidSPAdes (for monocultured bacterial genomes) and Recycler (for metagenomes) may be complementary methods for plasmid reconstruction [141]. Two studies have compared the performances of the four plasmid analysis tools (cBAR, PlasmidFinder, plasmidSPAdes, and Recycler) [148,149].

## 4 Concluding Remarks

Plasmids have been used as primal genetic tools for microbial engineering, particularly for nonmodel organisms. In synthetic biology, there have been attempts to build a vector by assembling functional modules [27,150]. Fortunately, the number of known plasmid sequences has increased dramatically in recent years, which has enabled us to detect core genes and co-evolving gene sets for each plasmid group. Plasmid functional modules identified by experimental or bioinformatics methods can contribute to biological parts/module databases, such as BioBrick [26], SEVA [27], and Clostron [150].

Following the rules of the natural plasmid genome, we can design synthetic plasmids. For example, a set of core genes as well as a selection marker or TA system should be included to prevent generation of plasmid-free cells (Table 2). The G + C content of a plasmid should be similar to (and lower than) that of the host, and highly expressed essential genes should be located on lagging strand templates. We also emphasize that optimization of external settings for the plasmid, for example, type of growth medium and presence or absence of spatial structure in the growth environment, could greatly influence plasmid population dynamics. Although further work is needed, a synthetic biology approach, e.g., de novo synthesis of artificial plasmids followed by experimental evaluation of plasmid maintenance, may lead to the construction of stable vectors and improve our understanding of why plasmids are so successful as genetic parasites.

## Competing Interests

The authors declare no competing interests.

## Authors' Contributions

Conceptualization: TO; data analysis: HS; resources: MT; writing – original draft preparation: HY, HS, MS, and TO; writing – review and editing: HY, HS, MS, and TO.

## Acknowledgements

We thank members of the Institute for Advanced Biosciences at Keio University for useful discussions. We also thank three anonymous reviewers for suggestions for improving this review. This work was supported in part by research funding from Keio University, Yamagata Prefecture, and Tsuruoka City to H.S. and M.T. and by Japan Society for the Promotion of Science (JSPS) KAKENHI (grant numbers 26106001 and 26450090 to T.O., 15H05618 to M.S., and 18K06357 to H.Y.). Computational resources were provided by the Data Integration and Analysis Facility, National Institute for Basic Biology. We would like to thank Editage (www.editage.jp) for English language editing.
